# Acute-serum amyloid A and A-SAA-derived peptides as formyl peptide receptor (FPR) 2 ligands

**DOI:** 10.3389/fendo.2023.1119227

**Published:** 2023-02-03

**Authors:** Sara Abouelasrar Salama, Mieke Gouwy, Jo Van Damme, Sofie Struyf

**Affiliations:** Laboratory of Molecular Immunology, Department of Microbiology, Immunology and Transplantation, Rega Institute for Medical Research, University of Leuven, Leuven, Belgium

**Keywords:** serum amyloid A, allosteric modulation, chemotaxis, post-translational modification (PTM), inflammation, drug target, G protein-coupled receptor (GPCR), formyl peptide receptor (FPR)

## Abstract

Originally, it was thought that a single serum amyloid A (SAA) protein was involved in amyloid A amyloidosis, but in fact, SAA represents a four‐membered family wherein SAA1 and SAA2 are acute phase proteins (A-SAA). SAA is highly conserved throughout evolution within a wide range of animal species suggestive of an important biological function. In fact, A-SAA has been linked to a number of divergent biological activities wherein a number of these functions are mediated *via* the G protein-coupled receptor (GPCR), formyl peptide receptor (FPR) 2. For instance, through the activation of FPR2, A-SAA has been described to regulate leukocyte activation, atherosclerosis, pathogen recognition, bone formation and cell survival. Moreover, A-SAA is subject to post-translational modification, primarily through proteolytic processing, generating a range of A-SAA-derived peptides. Although very little is known regarding the biological effect of A-SAA-derived peptides, they have been shown to promote neutrophil and monocyte migration through FPR2 activation *via* synergy with other GPCR ligands namely, the chemokines CXCL8 and CCL3, respectively. Within this review, we provide a detailed analysis of the FPR2-mediated functions of A-SAA. Moreover, we discuss the potential role of A-SAA-derived peptides as allosteric modulators of FPR2.

## 1 Introduction

Maintaining homeostasis is essential for the health of biological systems. Indeed, both primordial and adaptable mechanisms exist to reestablish homeostasis following disturbance. An example of the former is the acute phase response (APR) which accompanies conditions such as trauma, infections, malignancies and immunological disorders. Characteristic of this primordial response are changes in the level of functionally diverse liver-derived plasma proteins known as acute phase proteins (APPs). Perhaps the most notorious of the APPs is serum amyloid A (SAA) (1). SAA constitutes a family of four proteins referred to as SAA1, SAA2, SAA3 and SAA4 of which SAA1 and SAA2 are of particular relevance. The latter SAAs are highly upregulated during the APR and are hence referred to as acute-SAAs (A-SAA) (2). In fact, A-SAA levels reportedly increase by as much as 1000-fold following activation of the APR (1). Murine A-SAAs are comprised of SAA1, SAA2 and SAA3; on the contrary, in humans *SAA3* is most often classified as a pseudogene. In humans and mice, SAA4 is constitutively produced. Whereas A-SAAs are also extra-hepatically expressed, the liver is regarded as their main source of expression (2). SAA1 and SAA2 display multiple polymorphic coding alleles (SAA1.1 to SAA1.5 and SAA2.1 to 2.2, respectively) encoding proteins that exhibit minor amino acid substitutions (3, 4) (**Table 1**). A‐SAAs function as apolipoproteins primarily binding high-density lipoprotein (HDL) in the circulation (5). A multitude of diverse biological functions have been attributed to A-SAAs (2) that are supposedly mediated through a variety of functionally distinct receptors including Toll-like receptor (TLR)2, TLR4, receptor for advanced glycation end-products (RAGE), P2X purinoceptor 7, CD36, scavenger receptor class B type 1 (SR-BI), its splice variant SR-BII, and formyl peptide receptor (FPR)2/formyl peptide receptor like (FPRL)1 (6). Nevertheless, the extent of A-SAA-mediated inflammatory functions and the promiscuity of their receptor profile have been under debate recently. Indeed, with respect to the TLR-mediated effects of A-SAA, it became recently evident that most if not all of these TLR-dependent activities are in fact mediated by lipopolysaccharide and/or lipoprotein contamination present in commercially available preparations of recombinant A-SAA (7–10). Similarly, it cannot be excluded that some FPR2-mediated effects of A-SAA that could not be confirmed in other laboratories are due to contaminating FPR2 ligands present in impure A-SAA preparations, such as formyl peptides. A major reason for this discrepancy lies in the usage of A-SAA from different sources, which display different degrees of purity. Nonetheless, whereas the TLR-mediated functions of A-SAA are now considered rather dubious, using rhSAA1.1 purified to homogeneity we recently confirmed that A-SAA is indeed an FPR2 ligand (7). However, it must be concluded that for many biological activities ascribed to A-SAA the literature is conflicting in that some effects observed in one laboratory could not be confirmed in another. To this end, we provide the reader with an overview of the available A-SAA preparations while indicating in the text which A-SAA source was used in each case. **Table 1** gives an overview of the different commercially available recombinant A-SAA preparations in comparison to the naturally occurring variants. In the present paper, we highlight and discuss A-SAA-FPR2 mediated functions. Moreover, we give insight into possible ways through which A-SAA and A-SAA-derived peptides may differentially activate FPR2. Finally, we discuss the potential role of A-SAA and its derived peptides as allosteric modulators of FPR2.

**Table 1:** Amino acid sequence of naturally occurring and commercially available recombinant human A-SAA preparations discussed in the present paper.

	Amino acid sequence
**Natural human SAA1.1**	RS FFSFL GEAFD GARDM WRAYS DMREA NYIGS DKYFH ARGNY DAAKR GPGGV WAAEA ISDAR ENIQR FFGHG AEDSL ADQAA NEWGR SGKDP NHFRP AGLPE KY
**Natural human SAA1.2**	RS FFSFL GEAFD GARDM WRAYS DMREA NYIGS DKYFH ARGNY DAAKR GPGGA WAAEV ISDAR ENIQR FFGHD AEDSL ADQAA NEWGR SGKDP NHFRP AGLPE KY
**Natural human SAA1.3**	RS FFSFL GEAFD GARDM WRAYS DMREA NYIGS DKYFH ARGNY DAAKR GPGGA WAAEA ISDAR ENIQR FFGHG AEDSL ADQAA NEWGR SGKDP NHFRP AGLPE KY
**Natural human SAA1.4**	RS FFSFL GEAFD GARDM WRAYS DMREA NYIGS DKYFH ARGNY DAAKR GPGGA WAAEV ISNAR ENIQR FFGHG AEDSL ADQAA NEWGR SGKDP NHFRP AGLPE KY
**Natural human SAA1.5**	RS FFSFL GEAFD GARDM WRAYS DMREA NYIGS DKYFH ARGNY DAAKR GPGGA WAAEV ISDAR ENIQR FFGHG AEDSL ADQAA NEWGR SGKDP NHFRP AGLPE KY
**Natural human SAA2.1**	RS FFSFL GEAFD GARDM WRAYS DMREA NYIGS DKYFH ARGNY DAAKR GPGGA WAAEV ISNAR ENIQR LTGHG AEDSL ADQAA NKWGR SGRDP NHFRP AGLPE KY
**Natural human SAA2.2**	RS FFSFL GEAFD GARDM WRAYS DMREA NYIGS DKYFH ARGNY DAAKR GPGGA WAAEV ISNAR ENIQR LTGRG AEDSL ADQAA NKWGR SGRDP NHFRP AGLPE KY
**Recombinant human SAA1.1 (rhSAA1.1; Peprotech^)^**	M^a^RS FFSFL GEAFD GARDM WRAYS DMREA NYIGS DKYFH ARGNY DAAKR GPGGV WAAEA ISDAR ENIQR FFGHG AEDSL ADQAA NEWGR SGKDP NHFRP AGLPE KY
^b^ **Hybrid recombinant human SAA (rhSAA; Peprotech)**	M^a^RS FFSFL GEAFD GARDM WRAYS DMREA NYIGS DKYFH ARGNY DAAKR GPGGV WAAEA ISNAR ENIQR FFGRG AEDSL ADQAA NEWGR SGKDP NHFRP AGLPE KY
^c^ **Eukarytoic recombinant human SAA1.1 (erhSAA1.1; Origene)**	RS FFSFL GEAFD GARDM WRAYS DMREA NYIGS DKYFH ARGNY DAAKR GPGGV WAAEA ISDAR ENIQR FFGHG AEDSL ADQAA NEWGR SGKDP NHFRP AGLPE KYEQK LISEE DL

a Methionine residue as a consequence of recombinant synthesis in Escherichia coli.

b A hybrid form of recombinantly expressed human SAA1.1 and SAA2.2 in which position 61 is occupied by asparagine (N) and position 72 is occupied by arginine (R) derived from SAA2.2 replacing the aspartic acid (D) and histidine (H) residues in SAA1.1, respectively.

c Human SAA1.1 recombinantly expressed in HEK293 cells containing a residual myc-tag at the carboxy-terminus.

FPR1 to 3 comprise a class of G protein-coupled receptors (GPCRs) that derive their nomenclature from their capacity to recognize formylated bacterial peptides. In comparison to other FPRs, FPR2 shows an expression that is more promiscuous. Indeed, FPR2 is reportedly expressed by a diverse spectrum of cells including phagocytic leukocytes, T-lymphocytes, endothelial cells, epithelial cells, neuroblastoma cells and hepatocytes to name a few (11). In line with other GPCRs, that respond to a wide array of physically and chemically distinct ligands (12), FPR2 is rather indiscriminate in the ligands that it recognizes. FPR2 ligands include microbe-derived peptides of viral and bacterial origin. Moreover, FPR2 recognizes endogenous mitochondrial peptides, amyloidogenic proteins (A-SAA, prion protein and neuroprotective peptide) and their derived peptides, peptides derived from regulators of inflammation (urokinase-type plasminogen activator, cathelicidin, CCL23, neuropeptide and pleiotropic peptide) and annexin A1 and annexin A1-derived peptides. Furthermore, FPR2 ligands extend to non-peptide molecules. This GPCR also recognizes lipids such as the immunoregulatory lipoxin A4 (LXA4). As such, FPR2 is implicated in a variety of pathophysiological functions including innate host defense, inflammatory responses in amyloidosis and neurodegenerative diseases and immunoregulation (13).

## 2 A-SAA-induced expression of FPR2

Besides activating FPR2 to mediate various pathophysiological functions (*vide infra*), A-SAA plays a role in regulating FPR2 expression through an unidentified receptor interaction. Indeed, the stimulation of naïve bone marrow-derived murine neutrophils with rhSAA1.1, led to an upregulation of FPR2 (14). Niu et al. demonstrated a similar upregulation of FPR2 following the treatment of peripheral blood neutrophils with rhSAA1.1 (15). However, it is currently unclear to what extent A-SAA is involved in modulating FPR2 expression. Nonetheless, the literature exhibits evidence demonstrating the co-expression of A-SAA and FPR2 within the context of inflammation. O’Hara et al. demonstrated the co-expression of A-SAA and FPR2 in synovial tissue derived from patients with variable arthropathies including rheumatoid arthritis (RA), sarcoid arthritis, psoriatic arthritis and undifferentiated arthritis (16). Moreover, increased co-expression of SAA1 and FPR2 was observed in neutrophils isolated from breast cancer patients in comparison to those derived from healthy controls (15). Furthermore, in a murine bacterial pneumonia model where mice were co-infected with *Streptococcus pneumoniae* and influenza A virus, SAA1 and FPR2 were markedly upregulated in comparison to mice treated with a single pathogen or saline (17). Finally, Ren et al. were the first to demonstrate the simultaneous ocular expression of SAA1, SAA3 and FPR2 mRNA following induced corneal neovascularization in mice (18). The purpose behind A-SAA-mediated FPR2 upregulation is unclear. Perhaps A-SAA upregulates the expression of FPR2 to aid in the host’s innate immune defense. Indeed, as the APR is activated during infection, an upregulation of FPR2 on leukocytes would lead to enhanced detection of microbial-derived FPR2 ligands following microbial infiltration. In contrast, enhanced FPR2 expression during the resolution stage of the disease may lead to a stronger pro-resolving response, as FPR2 recognizes pro-resolving molecules such as LXA4 (13).

## 3 Pathophysiological roles mediated *via* the FPR2-SAA interaction

### 3.1 Chemotaxis

In 1994 Badolato et al. were the first to describe a dose-dependent chemotactic effect of rhSAA on human polymorphonuclear leukocytes (PMNs) and peripheral blood mononuclear cells (PBMCs) both *in vitro* and *in vivo*. Moreover, rhSAA upregulated the expression of cell adhesion molecules including CD18/CD11b and CD18/CD11c. This finding was further complemented by adhesion assays that demonstrated increased adhesion of rhSAA-treated PMNs and PBMCs to endothelial cells (19). A year later, the same group demonstrated the inhibition of rhSAA-mediated human monocyte chemotaxis following pertussis toxin (PTx) treatment (20). While this narrowed down the receptor utilized by rhSAA during chemotaxis to a GPCR, it was only in 1999 that rhSAA was demonstrated to utilize FPR2 to chemoattract leukocytes (21). The *in vitro* recruitment of human neutrophils by rhSAA *via* FPR2 activation was later corroborated by Lee et al. (22). In addition, the chemoattractant effect of rhSAA on murine neutrophils has been demonstrated to occur *via* the murine counterpart of human FPR2 (23). In line with this, FRP2^-^/^-^ mice show reduced overall cellular trafficking in an interleukin (IL)-1β-induced air pouch model following intravenous administration of rhSAA (24). Moreover, Chen et al. demonstrated the role of adipocyte-derived murine SAA3 in the recruitment of bone marrow-derived macrophages *via* FPR2 within the context of obesity (25). Over the years, the chemotactic effect of A-SAA has expanded to include more cell types such as mast cells, CD14^+^ monocytes, immature dendritic cells, macrophages, T-lymphocytes, smooth muscle cells, an epithelial ovarian cancer cell line (SKOV3) and endothelial cells (24, 26–31).

We recently provided additional evidence on the utilization of FPR2 by rhSAA1.1 during *in vitro* neutrophil chemotaxis using the selective FPR2 antagonist WRW4 (32). However, as previously discussed, a number of published data investigating the biological activity of recombinantly expressed SAA might in fact be mediated by contaminating bacterial antigens rather than being inherent to the APP itself (*vide supra*). This led us to question whether the FPR2-dependent chemotactic function of A-SAA might be mediated *via* contaminating formylated bacterial peptides. To this end, we investigated the *in vivo* chemotactic potential of rhSAA1.1 purified to homogeneity, *via* reversed-phase high-performance liquid chromatography, in C57BL/6 mice. We observed significant recruitment of neutrophils and mononuclear cells into the joint upon intra-articular injection of the purified rhSAA1.1, thereby solidifying the FPR2-mediated chemotactic function of A-SAA (7).

Synergy between GPCR ligands has been described within the framework of leukocyte chemotaxis. Indeed, chemokines have been shown to synergize not only with each other but also with other non-chemokine GPCR inflammatory ligands (33). The latter phenomenon is of interest in relation to A-SAA. Much to our surprise, we observed a synergistic effect between rhSAA1.1 and CXCL8 in the activation and recruitment of human neutrophils evidenced through neutrophil shape change and cell migration in the Boyden chamber chemotaxis assay, respectively (32). De Buck et al. recently purified a supposed monocyte chemotactic factor from bovine serum, which was later identified as a C-terminal fragment of SAA1 corresponding to the amino acids 46-122 of the mature protein. Interestingly, while this C-terminal fragment of bovine SAA1 lacked the capacity to induce human leukocyte recruitment as a single inducer, it synergized with CXCL8 and CCL3 in the recruitment of neutrophils and CD14^+^ monocytes, respectively. This synergistic effect was dependent upon FPR2 activation. Moreover, this bovine fragment could also synergize with murine CXCL6 *in vivo* to enhance the intraperitoneal recruitment of neutrophils in NMRI mice (34). In a similar manner, rhSAA1.1 peptides generated by matrix metalloproteinase (MMP)-9 corresponding to the amino acids 52-104 and 58-104 of SAA1.1 possess the capacity to synergize with CXCL8 in the activation and recruitment of human neutrophils, a finding that was not shared by their N-terminal counterpart, SAA1.1(1–51) (35).

### 3.2 Induction of inflammatory modulators

#### 3.2.1 Cytokines and chemokines

To mount an effective immune response, a mechanism by which the different leukocytes types involved can communicate is necessary. This role is fulfilled by cytokines. Indeed, cytokines are a large and broad class of proteins that play a major role in regulating the immune response. One major cytokine subclass is the family of chemotactic cytokines “chemokines”, which play a specialized and cardinal role through their regulation of cellular traffic throughout the body (36). Current literature exhibits evidence indicating the involvement of FPR2 in A-SAA-induced cytokine and chemokine expression. Indeed, He et al. demonstrated upregulation of the prototypical neutrophil chemoattractant CXCL8 following a 4-hour treatment of human neutrophils with rhSAA, where CXCL8 expression was shown to occur in a *de novo* manner. Moreover, using an antibody directed against FPR2, the authors could neutralize CXCL8 expression in response to rhSAA (37). Furthermore, FPR2-mediated induction of CCL2, a monocyte chemoattractant, by rhSAA was also demonstrated on human monocytes (38). The FPR2-mediated inductive capacity of rhSAA has also been demonstrated on human umbilical vein endothelial cells (HUVECs), where an upregulated expression of CCL2 in HUVECs was observed after the cells were cultured with rhSAA for 24 hours. Knockdown experiments demonstrated that the expression of CCL2 by HUVECs in response to rhSAA was mediated by FPR2, but in a PTx-insensitive manner and involved the p38 mitogen-activated protein kinase (MAPK) and Jun N-terminal kinase (JNK) signaling pathways (39). In keeping with umbilical cord endothelial cells, the treatment of human carotid artery endothelial cells with the FPR2 antagonist WRW4 elicited an inhibitory effect on the nuclear factor (NF)-κB-mediated tumor necrosis factor (TNF)-α -inductive capacity of rhSAA (40).

Besides the inductive capacity on the aforementioned cell types, rhSAA1.1-induced cytokine expression has also been demonstrated in primary murine microglial cells. Following a 4-hour treatment with rhSAA1.1, microglial cells displayed upregulated gene expression of TNF-α, IL-1β, IL-6 and CCL2, that was neutralized by the pro-resolving FPR2 agonist aspirin-triggered-resolvin D1 (AT-RvD1). However, it is unclear whether the inhibitory effect of AT-RvD1 on rhSAA1.1-induced cytokine expression is due to receptor competition or rather a general anti-inflammatory effect brought about by AT-RvD1 (41). Bozinovski et al. demonstrated the role of A-SAA in cytokine induction within the context of chronic obstructive pulmonary disease (COPD). Following the treatment of the lung epithelial cell line A549 (deficient for FPR2) with rhSAA, there was no significant expression of CCL2 and granulocyte-macrophage colony-stimulating factor (GM-CSF). On the contrary, A549 cells transfected to express FPR2 showed a marked increase in CCL2 and GM-CSF expression in response to rhSAA stimulation. Moreover, pretreatment of FPR2-transfected A549 cells with lipoxin A4 (LXA4) and 15-epi-LXA4 reduced rhSAA-induced CXCL8 expression, an effect which the authors attribute to competitive antagonism (42) or alternatively could be caused by reduced FPR2 at the cell surface due to receptor internalization following activation of FPR2 by LXA4 or 15-epi-LXA4 (43).

Having said that, the aforementioned reports are in contrast to what was observed in our hands. Whereas rhSAA1.1 induced CXCL8 expression in human CD14^+^ monocytes, the treatment of human neutrophils with rhSAA1.1 failed to induce the expression of CCL3 and CXCL8 following an incubation period of up to 24 hours (32). Later on, rhSAA1.1 that had been purified to homogeneity failed to provoke chemokine expression in CD14^+^ monocytes (7). Commiserating with our data, Christenson et al. showed a lack of increased plasma inflammatory cytokines in response to elevated plasma A-SAA in RA patients. Moreover, transgenic mice in which adipocytes overexpressed human SAA1 failed to show increased plasma levels of the murine neutrophil chemoattractant, CXCL1 (10). Furthermore, Burgess et al. demonstrated that eukaryotic rhSAA1.1 (**Table 1**) expressed in mammalian human embryonic kidney 293T cells did not induce the expression of inflammatory cytokines in human PBMCs (9).

#### 3.2.2 Matrix metalloproteinases (MMPs)

MMPs comprise a class of proteases with the main function of processing extracellular matrix proteins, thereby implicating MMPs in various diverse functions such as bone growth and remodeling, angiogenesis, wound healing and cell migration (44). Lee et al. demonstrated the expression of MMP-9 in monocytic THP-1 cells following stimulation with rhSAA for 24 hours. SAA-provoked MMP-9 expression co-occurred with an increase in intracellular calcium levels and was PTx-sensitive. Furthermore, using FPR2/MMP-9 luciferase transfected Chinese hamster ovary (CHO) cells, the authors demonstrated the utilization of FPR2 by rhSAA in the expression of MMP-9. Moreover, the authors confirmed the expression of MMP-9 in a relatively more physiological setting through the stimulation of human peripheral blood monocytes with rhSAA (45). In contrast, but similar to what was observed concerning chemokine induction, we observed a lack of MMP-9 expression by human CD14^+^ monocytes following stimulation with rhSAA1.1 that had been purified to homogeneity (7).

#### 3.2.3 Acute phase proteins (APPs)

The pentraxins form a highly conserved family of proteins comprised of C-reactive protein, serum amyloid P and pentraxin 3 (PTX3). Similar to A-SAA, pentraxins are also regarded as APPs. Of particular relevance is PTX3, which is thought to play a role in regulating host-mediated immune responses (46). In a study by Satomura et al., rhSAA was shown to upregulate both the mRNA and protein expression of PTX3 in RA-derived synovial fibroblasts, wherein the addition of HDL mitigated the PTX3-inductive capacity of rhSAA. rhSAA was also found to upregulate PTX3 expression in osteoarthritis (OA)-derived synovial fibroblasts albeit to a lesser extent than that observed on RA-derived synovial fibroblasts. Moreover, rhSAA-induced upregulation of PTX3 was mediated *via* the NF-κB, p38 JNK and MAPK signaling pathways. To determine the receptor utilized by rhSAA during this APP induction, synovial fibroblasts were nucleofected with FPR2 siRNA after which the capacity of rhSAA to induce PTX3 was significantly downregulated (47). Analogously, the treatment of human aortic endothelial cells (HAECs) with rhSAA instigated the release of PTX3 on both mRNA and protein level, and this could be inhibited by the selective FPR2 antagonist WRW4 and FPR2 siRNA, further cementing the role of FPR2 in rhSAA-mediated PTX3 induction (48). Similar to the findings of Satomura et al., the authors demonstrated that rhSAA required the transcription factor NF-kB and the JNK signaling pathway to induce the expression of PTX3 (48).

### 3.3 Formation of atherosclerotic plaques

While A-SAA is largely regarded as an atherosclerosis disease biomarker (49), growing evidence suggests that SAA is not merely a passive participant but rather plays an active role in the formation of atherosclerotic plaques through the modulation of several disease-related pathways. The contribution of A-SAA to atherosclerotic plaque formation *via* FPR2 activation is discussed below.

#### 3.3.1 Foam cell formation

The formation of macrophage-derived foam cells is a hallmark of atherosclerosis pathology. Foam cells are formed following the infiltration and accumulation of macrophages into the arterial intima media where macrophages display uncontrolled uptake of oxidized-low-density lipoproteins (LDL) and impaired cholesterol release leading to the formation of cytoplasmic lipid droplets and hence the formation of foam cells (50). Whereas macrophages are the main cells involved in foam cell formation, these fat-laden cells may also derive from endothelial cells and vascular smooth muscle cells (51). The A-SAA-FPR2 interaction has been implicated in macrophage foam cell formation. Indeed, following a 24-hour incubation period with rhSAA, RAW264.1 macrophages showed increased oil red O staining, which is characteristic of foam cells. Moreover, cells treated with rhSAA showed an upregulation of the lectin-like oxidized LDL receptor 1 (LOX-1). The formation of foam cells following treatment with rhSAA was inhibited by the selective FPR2 antagonist WRW4 and by FPR2 siRNA. To further elucidate the signaling pathway utilized by rhSAA in foam cell formation, the authors treated rhSAA-stimulated RAW 264.1 cells with PTx. The aforementioned experiment demonstrated that rhSAA-induced foam cell formation is mediated through a PTx-independent signaling pathway. More specifically, JNK phosphorylation was deemed necessary for this cellular transformation (52).

#### 3.3.2 Induction of lipoprotein-associated phospholipase A_2_ (Lp-PLA_2_)

Lp-PLA_2_, is also known as platelet-activating factor acetylhydrolase owing to its capacity to degrade platelet-activating factor. This lipase is associated with lipoproteins in the plasma and particularly hydrolyzes circulating oxidized phospholipids involved in oxidative stress. Lp-PLA_2_ is highly implicated in atherogenesis and seems to play a role in every stage of atherosclerosis (53). Li et al. investigated the expression of Lp-PLA_2_ in response to rhSAA1.1 stimulation and observed an increased expression of Lp-PLA_2_ mRNA and protein in human monocytic THP-1 cells. Similar findings were observed in circulating human monocytes and the human macrophage cell line U937. The pre-treatment of THP-1 cells with the FPR2 antagonist WRW4 brought about a dose-dependent decline in Lp-PLA_2_ expression following rhSAA1.1 treatment. Furthermore, FPR2 signaling was required for peroxisome proliferator-activated receptor (PPAR)-γ activation and JNK, extracellular signal-regulated kinase (ERK)1/2 and p38 phosphorylation that lead to Lp-PLA_2_ expression. Lentiviral expression of murine SAA1 in atherosclerosis-prone ApoE^-^/^-^ mice led to an elevation of Lp-PLA_2_ co-localizing with macrophages in atherosclerotic plaques (54).

#### 3.3.3 Induction of Visfatin

Levels of circulating Visfatin, an adipocytokine, have been proposed to correlate with the extent of atherosclerosis and hence might be considered to function as a disease biomarker. A multitude of biological activities are attributed to the adipocytokine amongst which are many that promote plaque formation (55). In an attempt to understand Visfatin regulation, Wang et al. investigated the expression of Visfatin in the macrophage cell line RAW264.7 and primary human monocytes. They observed an upregulation of Visfatin in response to rhSAA1.1. Similar to the expression of Lp-PLA_2_, rhSAA1.1 utilized FPR2 to induce Visfatin *via* PPAR-γ signaling and ERK1/2 phosphorylation (56). Li et al. corroborated the finding that rhSAA activates PPAR-**γ** signaling *via* FPR2 activation, in the hepatocellular carcinoma cell line HepG2 (57).

#### 3.3.4 Vascular proteoglycan synthesis

Wilson et al. provided evidence for the implication of A-SAA in proteoglycan synthesis within the context of atherosclerosis. Following the treatment of monkey vascular smooth muscle cells with recombinant SAA (rSAA; unknown origin); an increase in sulphate incorporation was observed, reflecting proteoglycan synthesis. Through ionic interactions, proteoglycans bind the positively charged residues on apolipoproteins *via* their negatively charged sulfate groups. Indeed, rSAA-induced proteoglycans showed increased LDL binding. Moreover, using electrophoresis, the authors could confirm reduced electrophoretic mobility of proteoglycans derived from rSAA-stimulated cells, indicative of increased glycosaminoglycan chain length, an effect that was neutralized through competitive binding of FPR2 using its agonist LXA4. Furthermore, the addition of LDL or HDL mitigated the effect of rSAA on proteoglycan synthesis suggesting that rSAA must be in its free form to promote this atherogenic function. Using an antibody directed against transforming growth factor (TGF)-β the effect of rSAA on proteoglycan synthesis could be neutralized demonstrating that rSAA-mediated proteoglycan synthesis is an indirect effect mediated *via* TGF-β upregulation. Finally, through the use of a recombinant SAA1 adenovirus, the authors showed the co-localization of SAA1, biglycan and apolipoprotein B in the aortic root of ApoE^-^/^-^ C57BL/6 mice (58).

### 3.4 Allergy

Through A-SAA (SAA1.1 and SAA2.1) knockout (KO) in C57BL/6 mice, Smole et al. demonstrated a reduced type 2 immune response following house dust mite (HDM) exposure. Indeed, A-SAA knock-out (KO) mice showed reduced serum immunoglobulin E concentrations, bronchoalveolar lavage (BAL) eosinophilia, and goblet cell metaplasia of the airway lumen in comparison to wild-type (WT) animals. This occurred in line with notably lower numbers of dendritic cells and type 2 innate lymphoid cells and reduced Th2-cytokine production. Epithelial cell-derived IL-33 is important for the expansion of type 2 innate lymphoid cells that play a role in maintaining the local allergic response. Interestingly, the KO of A-SAA in C57BL/6 mice challenged with HDM brought about no change in baseline IL-33 levels in BAL fluid. On the contrary, WT mice showed a significant increase in IL-33 expression in BAL fluid upon exposure to HDM. Further *in vitro* experiments provided evidence of the mechanism by which A-SAA regulates the local allergic response. Indeed, HDM treatment of an airway epithelial cell line, BEAS-2B, concurrent with siRNA inhibiting SAA1, reduced expression of IL-33. Moreover, SAA1 was shown to bind to mite allergens Der p 13 and Blo t 13 upon which the formed complex activated FPR2 leading to the expression of IL-33 in the BEAS-2B cell line (59).

### 3.5 Regulation of apoptosis

The FPR2-mediated anti-apoptotic function of rhSAA was initially demonstrated within the context of arthritic diseases, where RA-derived and OA-derived fibroblast-like synoviocytes (FLS) showed increased proliferation, following treatment with rhSAA. This proliferative effect was reduced upon the introduction of FPR2 siRNA. This was further complemented by MTT and DNA fragmentation assays, which illustrated the anti-apoptotic effect of rhSAA in response to serum-free starvation. Moreover, rhSAA protected against sodium nitroprusside-, nitric oxide- or anti-Fas IgM plus cycloheximide-induced apoptosis (60). In line with the aforementioned findings, García et al. demonstrated an anti-apoptotic effect of rSAA (unknown origin) on neutrophils. Indeed, the treatment of neutrophils with rSAA brought about an increase in the percentage of viable neutrophils relative to the DMSO control while decreasing the percentage of apoptotic and dead neutrophils. The anti-apoptotic effect of rhSAA1.1 extends beyond neutrophils to include monocytes. We have observed enhanced survival of monocytes following treatment with homogenously purified rhSAA1.1 (7). By delaying the apoptosis of neutrophils and monocytes, A-SAA prolongs their lifespan and contributes to the inflammatory response. In contrast, the pre-treatment of neutrophils with the FPR2 agonist BMS-986235 reduced the percentage of viable neutrophils and increased the percentage of apoptotic cells in response to rhSAA1.1, thereby implicating FPR2 in the rSAA-mediated anti-apoptotic effect (61). Thus, activation of FPR2 with pro-resolution agonists such as BMS-986235 or endogenous molecules such as 15-epi-LXA4 can override the anti-apoptotic signal from A-SAA and drive resolution (62).

### 3.6 Macrophage polarization

Li et al. demonstrated the capacity of A-SAA to regulate macrophage phenotype within the context of hepatocellular carcinoma (HCC) in a pleiotropic manner. The treatment of U937 macrophages with rSAA (unknown origin) skewed the phenotype towards an M2-like subset characterized by IL-10^high^, IL-12p35^low^ and IL-23p19^high^ an effect that was mitigated by the FPR2 antagonist WRW4. Moreover, rSAA-treated macrophages showed an upregulation of IL-1β, IL-6, TNF-α and CXCL8. *In vivo*, LL-37, an FPR2 ligand, provoked a macrophage phenotype similar to that induced by rSAA *in vitro*. Indeed, analysis of tumor infiltrating macrophages from *in situ* tumor tissue following LL-37 treatment showed macrophages characterized by high IL-10 and low IL-12p35 expression levels, thereby implicating FPR2 signaling in regulating the phenotype of tumor-associated macrophages. In a paracrine manner, through its action on HCC cell lines, namely murine H22 and human HepG2, rSAA upregulated the expression of macrophage-colony stimulating factor (M-CSF) and CCL2, which in turn regulate macrophage phenotype, contributing to the tumor-associated macrophage population. Indeed, the treatment of U937 macrophages in conjugation with M-CSF and CCL2 skewed the population towards an M2-like phenotype characterized by high IL-10 and low IL-12p35 expression. Simultaneous treatment with M-CSF and CCL2 also provoked the expression of IL-1β, IL-6, TNF-α, CCL17, CXCL8 and CXCL13. The cascade of reactive oxygen species (ROS)-MAPK (ERK and p38)-NF-kB-mediated signaling was shown to be involved in the upregulation of M-CSF and CCL2 by rSAA in HCC cell lines. Furthermore, through FPR2 knockdown, using short-hairpin RNA, the authors demonstrated the involvement of FPR2 in rSAA-induced M-CSF and CCL2 expression in HCC cell lines (63). On the contrary, we recently demonstrated that pure rhSAA1.1 as a single stimulus does not modulate the macrophage phenotype *in vitro* (7). Nonetheless, Wang et al. demonstrated, through *in vivo* application of a specific anti-SAA antibody, modulation of macrophage polarization by A-SAA during hepatic inflammation in mice (64). The data of Wang et al. and Li et al. suggest that SAA might indirectly regulate macrophage polarization either through induction of immune modulators or through its cooperation with other immune modulators, as has been reported for interferon (IFN)-γ and LPS in the induction of M1 macrophages. Alternatively, SAA may exert a priming effect thereby augmenting the action of immune modulators. This has been described for granulocyte-macrophage colony-stimulating factor (GM-CSF) and M-CSF which prime macrophages towards an M1 or M2 phenotype, respectively (65).

### 3.7 Bone formation

Parathyroid hormone (PTH) regulates bone remodeling *via* its GPCR, parathyroid hormone 1 receptor (PTH1R), expressed on the surface of osteoblasts and osteoclasts, thereby playing a regulatory role in bone formation and resorption, respectively. Through its activation of PTH1R, PTH stimulates bone anabolism *via* cyclic adenosine monophosphate (cAMP) signaling. Conversely, to promote bone catabolism, PTH acts on osteoblastic lineage cells upregulating the expression of receptor activator of nuclear factor kappa-B ligand (RANKL) which promotes osteoclast differentiation from hematopoietic precursor cells (66). In the presence of Cox2, RANKL-treated bone marrow macrophages (BMMs) secrete a factor that impedes PTH-induced cAMP-mediated differentiation of primary osteoblasts (67). To further elaborate on the mechanism of RANKL-mediated bone resorption, Choudhary et al. revealed murine SAA3 as the PTH inhibitory factor. Indeed, WT RANKL-treated BMMs displayed a marked upregulation of SAA3 expression in a prostaglandin E_2_ (PGE_2_)-dependent manner, an effect that was not observed by Cox2 KO BMMs. To corroborate the role of SAA3 in impeding PTH-induced bone anabolism, SAA3 expression in BMMs was knocked down using short hairpin RNA, upon which the stimulatory effect of PTH on cAMP in primary osteoblasts was restored. Furthermore, to elucidate the receptor utilized by SAA during this process, the authors could reverse the inhibitory effect of rhSAA on PTH in primary osteoblasts using the selective FPR2 antagonist WRW4 (68).

### 3.8 Pulmonary disease

Anthony et al. demonstrated a correlation between the number of infiltrating neutrophils and the degree of SAA staining in lung tissue derived from COPD patients undergoing treatment for solitary peripheral carcinoma. Using an animal model where Balb/c mice were subjected to chronic intranasal administration of rhSAA, a direct relationship between rhSAA levels and pulmonary neutrophil infiltration was demonstrated. Furthermore, 6 hours following rhSAA administration, the levels of T-helper (Th) 17-polarizing cytokines namely IL-6, IL-1β and IL-23 peaked which was synonymous with an upregulated expression of IL-17A. The main cellular source of rhSAA-induced IL-17A were CD4^+^ T-cells, γδ T-cells, and Epcam^+^ CD45^−^ epithelial cells. To elaborate on the pathway leading to neutrophil recruitment, mice were treated with a neutralizing antibody against IL-17A, whereupon a downregulation of the murine neutrophil chemoattractant CXCL1 was observed. This occurred in congruence with reduced pulmonary neutrophil infiltration. Furthermore, the administration of 15-epi-LXA_4_ could avert the inflammatory response mediated by rhSAA, thereby suggesting the involvement of FPR2 in the A-SAA-IL-17A axis (69). Following up on these findings Bozinovski et al. proposed that FPR2 could be a potential therapeutic target in the treatment of COPD (70).

### 3.9 Cancer

Upregulated expression of A-SAA has been documented in tumors of varying origins including uterine carcinoma, lung cancer and nasopharyngeal carcinoma (71–73). In fact, tumor cells serve as a source of A-SAA during early carcinogenesis when the APR has not yet been activated (74). Moreover, a meta-analysis demonstrated a link between elevated A-SAA levels and diminished disease-free survival, progression-free survival and overall survival in patients suffering from distinct solid tumors (75). Multiple studies have pointed towards the role of A-SAA in various cancer-promoting functions. A-SAA was shown to contribute towards colitis-associated cancer in mice through enhanced inflammatory cytokine expression and macrophage infiltration (76). In addition, the role of SAA1 in pancreatic cancer was demonstrated wherein the knockdown of SAA1 in a human pancreatic cell line (PANC-1) improved response to chemotherapy, reduced epithelial-to-mesenchymal transition and reduced invasive capacity (77). Moreover, in a recent review, Fourie et al. discussed the contribution of SAA to breast cancer pathology *via* its role in inflammasome activation (78). However, it is currently unclear which receptor is utilized by A-SAA during some of these cancer-promoting functions. Nonetheless, FPR2 is a potential suspect. Indeed, FPR2 has been linked to multiple cancers (79–82) and has even been discussed as a potential therapeutic target (83).

## 4 Post-translational modification (PTM) of A-SAA

### 4.1 Proteolytic processing of A-SAA

PTM of inflammatory proteins serves as a way to modulate the instigated response. In particular, proteolytic processing is of relevance within an inflammatory setting. One such example is the activation of IL-1. Indeed, IL-1 is expressed as a precursor protein, which needs to undergo proteolytic processing to generate the active endogenous pyrogen (5, 84). On the other hand, the proteolytic processing of chemokines, which are not expressed as precursor proteins, serves to modulate their biological activity leading to divergent outcomes on their function (85). In comparison to chemokines, the study of PTM of A-SAA is still in its infancy with only a few reports on its processing. Stix et al. were the first to reveal the susceptibility of plasma-derived A-SAA to undergo proteolytic processing by MMPs, namely MMP-1, -2 and -3 which cleave intact A-SAA at multiple positions (86). Further studies by van der Hilst et al. demonstrated the differential capacity of MMP-1 to cleave different A-SAA isoforms, where rhSAA1.5 was observed to be more resistant to proteolytic processing by MMP-1 in comparison to rhSAA1.1. The cleavage sites reported by van der Hilst et al. were similar to those described by Stix et al. (87). We recently demonstrated the susceptibility of rhSAA1.1 to undergo proteolytic processing by MMP-9, which shared cleavage sites with MMP-1, -2 and -3 in this APP (35). Moreover, the processing of rhSAA by cathepsins B and L has been evidenced by Röken et al. Interestingly, those proteases produced highly distinct rhSAA fragments. In fact, cathepsin B was found to possess endoproteolytic, carboxypeptidase and minor aminopeptidase activity; whereas, cathepsin L was shown to possess only endoprotease activity (88). Besides cathepsins B and L, cathepsin D also cleaved rhSAA1.1. Furthermore, the incubation of rhSAA1.1 with a splenic extract derived from RA patients induced N-terminal processing which was inhibited following the addition of the aspartate protease inhibitor pepstatin. As such, an aspartate protease, presumed to be cathepsin D, was implicated in this amino-terminus processing of A-SAA (89). Moreover, the capacity of elastase to clip rhSAA1.1 was also demonstrated. Elastase processed rhSAA1.1 at its N- and C-terminus leading to the generation of a range of different peptides (90). **Table 2** gives an overview of the A-SAA-derived cleavage products generated following incubation with proteases.

**Table 2:** Cleavage products of commercial and naturally occurring A-SAA variants following proteolytic cleavage by different proteases.

Protease	SAA source	Detected peptides^a^			Reference
MMP-1	rhSAA1.1	1-57	58-104		(87)
	rhSAA1.5	24-104	30-104	58-104	(87)
	Plasma-derived A-SAA	1-57	7-29,8-30 or 9-31	58-104	(86)
MMP-2	rhSAA1.1			52-104	(34)
	Plasma-derived A-SAA	1-51	8-55	52-104	(86)
MMP-3	Plasma-derived A-SAA	8-55 74-104	58-104	57-104	(86)
MMP-9	rhSAA1.1	1-51	52-104	58-104	(35)
Cathepsin B	rhSAA	1-99 1-85 1-78 1-69 3-52 3-42 4-42 4-27 5-25 6-25	1-87 1-83 1-76 1-67 3-50 3-31 4-31 4-20 5-22 6-20	1-86 1-82 1-74 2-69 3-47 4-51 4-26 5-46 6-26 7-20	(88)
Cathepsin D	rhSAA1.1	7-104	21-104		(89)
Cathepsin L	rhSAA	2-24 9-37 71-104	8-36 9-36	9-31 70-104	(88)
Splenic extract	rhSAA1.1	10-104	13-104	16-104	(89)
Elastase	rhSAA1.1	28-104	59-104	66-104	(90)

**^a^** Numbering is based upon the mature protein.

### 4.2 Natural occurrence of modified A-SAA variants

The initial discovery of SAA was in fact evoked by the identification of amyloid A protein, an N-terminal A-SAA-derived fragment involved in the formation of amyloid A plaques, characteristic of amyloid A amyloidosis, providing early evidence that A-SAA undergoes PTM (91). In line with this, modified A-SAA variants have been detected in patients suffering from diseases with variable underlying inflammatory etiologies. Early studies by Migita et al. utilized an immunoblot analysis approach to discern modified A-SAA in sera derived from RA patients. In addition to the full-length protein, two distinct A-SAA peptides, a 6.0-kDa and a 4.5-kDa peptide were detected. Interestingly, the ratio of abundancy of SAA-derived fragments to total SAA was higher in patients suffering from amyloidosis (92). Yassine et al. characterized the PTM of A-SAA polymorphic variants in the plasma of type 2 diabetes mellitus patients. Removal of the N-terminal arginine or the arginine-serine dipeptide resulting in SAA1.1(2-104 and 3-104), SAA1.3(2-104 and 3-104) and SAA2.1(2-104 and 3-104) was detected. The ratio of abundancy to the intact variant was lower in diabetics in comparison to non-diabetics (**Table 3**) (93). Furthermore, Tolson et al. identified three N-terminally processed variants of SAA1 including SAA1.1(2–104), SAA1.1(3-104) and SAA1.1(5-104) in the sera of renal carcinoma patients. Contrary to the observations made by Yassine et al., Tolson et al. did not detect N-terminally truncated A-SAA variants in healthy controls (94). Furthermore, Baba et al. described N-terminal truncation of A-SAA in non-amyloidogenic patients with an activated APR suggesting that this PTM is not unique to a particular disease aetiology (102). Also Trechevska and colleagues detected, N-terminal truncation of A-SAA in healthy populations, wherein they reported the truncation of up to five N-terminal amino acids of SAA1.1, SAA1.3, SAA2.1 and SAA2.2, thereby indicating that N-terminal processing of A-SAA occurs outside the context of disease (95). Besides N-terminal truncation, there is growing evidence indicating the occurrence of A-SAA-derived C-terminal peptides under inflammatory conditions. SAA1(92–104) was identified as a prognostic marker in the sera of renal carcinoma patients by Wood et al. (97). Furthermore, SAA1(98–104) has been detected in the synovial fluid of RA patients (98). SAA1(34-104) has been detected in the plasma of patients suffering from Kawasaki disease. Although detected in patients with the subacute disease, plasma levels of SAA1(34-104) were higher in patients at the acute stage of Kawasaki disease and the peptide was no longer detectable upon resolution of the disease (96). Evidence supporting the *in vivo* occurrence of A-SAA C-terminal peptides has also been reported in cows. De Buck et al. recently isolated bovine SAA1(46-112) from fetal calf serum, based upon biological activity, and subsequently characterized the bovine fragment (34).

**Table 3:** Natural occurrence, processing enzymes and biological activity of reported A-SAA-derived peptides.

A-SAA variant^a^	Detection method	*In vivo* occurrence	Disease (tissue)	Generating protease	Biological activity	Reference
SAA1.1 (2-104) SAA1.1 (3-104) SAA1.3 (2-104) SAA1.3 (3-104) SAA2.1 (2-104) SAA2.1 (3-104)	MALDI-TOF-MSIA-mass spectrometry	Yes	Type-II diabetes (plasma) Healthy population (plasma)	ND	ND	(93)
SAA1.1 (2-104) SAA1.1 (3-104) SAA1.1 (5-104)	ProteinChip-SELDI-TOF- mass spectrometry	Yes	Renal carcinoma (serum)	ND	ND	(94)
SAA1.1 (2-104) SAA1.1 (3-104) SAA1.1 (5-104) SAA1.1 (6-104) SAA1.3 (2-104) SAA1.3 (3-104) SAA2.1 (2-104) SAA2.1 (3-104) SAA2.1 (5-104) SAA2.2 (2-104) SAA2.2 (3-104)	MALDI-TOF-MSIA-mass spectrometry	Yes	Healthy population (plasma)	ND	ND	(95)
A-SAA (34-104)	ProteinChip-SELDI-TOF- mass spectrometry	Yes	Kawasaki disease (plasma)	ND	ND	(96)
A-SAA (92-104)	ProteinChip-SELDI-TOF- mass spectrometry	Yes	Renal carcinoma (serum)	ND	ND	(97)
A-SAA (98-104)	HPLC-ESI-mass spectrometry	Yes	Rheumatoid arthritis (synovial fluid)	ND	IFN-γ induction by T-cells *in vitro* Inhibition of SO production by neutrophils *in vitro* Inhibition of neutrophil chemotaxis *in vitro*	(98)
SAA1.1 (52-104)	NA	ND	NA	MMP-2 MMP-9	*In vitro* synergy with CXCL8 in neutrophil recruitment	(34, 35, 86)
SAA1.1 (58-104)	NA	ND	NA	MMP-1 MMP-3 MMP-9	*In vitro* synergy with CXCL8 in neutrophil recruitment	(35, 86, 87)
SAA1.1 (47-104)	NA	ND	NA	ND	*In vitro* synergy with CXCL8 in neutrophil recruitment	(34)
SAA1 (11-58) SAA1 (11-68) SAA1 (11-72) SAA1 (27-72) SAA1 (27-90) SAA1 (29-104)	NA	ND	NA	ND	*In vitro* and *in vivo* neutralization of LPS activity	(99)
A-SAA (1-14) A-SAA (15-104) A-SAA (83-104)	NA	ND	NA	ND	*In vitro* inhibition of fMLP-induced neutrophil activation	(100)
SAA1 (1-5)	NA	ND	NA	ND	Destabilization of SAA amyloid fibrils	(101)
SAA1 (46-112)^b^	HPLC-ESI-mass spectrometry	Yes	ND	ND	*In vitro* synergy with CXCL8 and CCL3 in neutrophil and monocyte recruitment, respectively *In vivo* synergy with murine CXCL6 in neutrophil recruitment	(34)

a A-SAA variants/polymorphic alleles are indicated as per indicated in the respective reference.

b The mentioned fragment was isolated from fetal calf serum.

CID, collision-induced dissociation; ESI, electrospray ionization; FAB, fast atom bombardment; HPLC, high-performance liquid chromatography; IFN, interferon; LC, liquid chromatography; MALDI, matrix-assisted laser desorption/ionization; MSIA, mass spectrometric immunoassay; NA, Not applicable; ND, Not determined; SELDI, surface enhanced laser desorption ionization; SO, superoxide; TOF, time of flight.

### 4.3 Biological activity of A-SAA peptides

The influence of naturally occurring N-terminal truncation on the biological activity of intact A-SAA has not yet been investigated; however, other A-SAA-derived peptides have been studied. In 1998, intact rhSAA and multiple synthetic SAA1-derived peptides were investigated for their modulatory effect on N-formylmethionyl-leucyl-phenylalanine (fMLP)-induced neutrophil functions. Both intact rhSAA and A-SAA(1-14) were found to inhibit fMLP-induced myeloperoxidase release and neutrophil-directed migration suggesting an anti-inflammatory effect relayed by SAA and its derived N-terminal peptide (100). Nonetheless, a year later, SAA was identified as an FPR2 ligand (21). As such, the inhibitory effect brought about by rhSAA and A-SAA1(1-14) on the FPR2-mediated fMLP functions is likely due to receptor desensitization, thereby suggesting that A-SAA1(1-14) might be a potential FPR2 ligand. More recently, the inhibitory effect of synthetic SAA1 fragments (29-104, 27-90, 27-72, 11-72, 11-68 and 11-58) on the LPS-mediated inflammatory response in macrophages was demonstrated (99). Furthermore, Yavin et al. demonstrated upregulated expression of IFN-γ following the stimulation of human T-lymphocytes with SAA1(98-104). Moreover, SAA1(98-104) bound to T-lymphocytes in a specific and saturable manner (98) (**Table 3**). Finally, A-SAA peptides play a role in chemotaxis by synergizing with chemokines to further enhance leukocyte recruitment to the site of inflammation (*vide supra*) (34, 35). Apart from the aforementioned studies, very little has been revealed regarding the biological activity conveyed by A-SAA proteolytic fragments.

## 5 Allosteric modulation of FPR2 by A-SAA and A-SAA-derived peptides: A potential therapeutic strategy

### 5.1 A-SAA, a presumed allosteric modulator of FPR2

Allosteric modulation of protein function was initially described in relation to enzymes and is now considered to play an integral role in the functionality of other proteins, among which are GPCRs (103). Allosteric ligands bind to active sites on receptors that are spatially and topographically distinct from those of the primary, orthosteric ligands. As such, the surface of GPCRs may reveal additional binding sites that have yet to be identified. Classically defined, an allosteric modulator is a ligand that does not play a role in receptor behavior in the absence of its orthosteric agonist, but rather serves to amplify the response generated by its orthosteric partner. On the contrary, an allosteric inhibitor locks the receptor in a conformation that inhibits the binding or the activity of its traditional orthosteric ligand (104). Nonetheless, allosteric agonists, molecules which can render a GPCR in its active state in the lack of the presence of an orthosteric ligand, have also been described (105). The nature of the interaction between A-SAA and FPR2 has been briefly investigated by Bena et al. (106). Using chimeric FPR2 clones, rhSAA was shown to engage extracellular loops 1 and 2 on FPR2 when inducing calcium fluxes. Extracellular loop 3 and the N-terminus of FPR2 were found to be dispensable for this SAA-mediated signaling (106). Recent cryogenic electron microscopy investigating the structure of the signaling complex of WKYMVm and FPR2 revealed the interaction of the peptide with extracellular loops 1 and 2 and the transmembrane domains 3-7 (107). Being a potent pan-agonist at FPR2, WKYMVm is regarded as an orthosteric FPR2 ligand (108–110). Although A-SAA and WKYMVm seem to share similar binding sites on FPR2, there is evidence to suggest that A-SAA does not bind the orthosteric binding site of FPR2. We and others have demonstrated the lack of competitive binding between A-SAA variants and WKYMVm (7, 45), as such, it is plausible that A-SAA functions as an allosteric FPR2 ligand.

In the late 1980s, circular-dichroism studies indicated that murine SAA2 and human SAA1 are helical in nature (111). In line with this, Lu and colleagues recently revealed the crystal structure of human SAA1.1, and showed that SAA1.1 is composed of a four-helix bundle fold stabilized by the C-terminal tail. In addition, the tendency of this A-SAA variant to form oligomers in solution, most notably a hexamer, was demonstrated; nonetheless, SAA1.1 was also found to exist as a monomer and dodecamer (112). Moreover, the capacity of murine SAA2.2 to exist in multiple oligomeric states, primarily as an octamer and a hexamer, has been demonstrated (113, 114). However, it is currently unknown whether A-SAA carries out its biological role as a monomer or rather through a higher oligomeric state. To render the matter more complex, human A-SAA and its murine counterpart have been described as intrinsically disordered proteins (IDP) (115, 116). Owing to their conformational plasticity, IDPs contain multiple motifs for interaction with their physiological partners, thereby allowing allosteric binding and regulation of distinct cellular signaling pathways (117). The structural instability of A-SAA may explain the capacity of this APP to instigate a wide array of biological functions. It is currently unknown whether the different oligomeric forms acquired by A-SAA play a role in the differential activation of FPR2. Moreover, since SAA is an IDP, the possibility that it may bind FPR2 in both its allosteric- and orthosteric-binding sites is not out of question. Indeed, the dual binding of FPR2 by A-SAA may explain the discrepancy observed in the literature regarding A-SAA-mediated functions. Indeed, Wold et al. described an alternative allosteric modulator-receptor complex instigating differential biology when compared to the native receptor (105). As such, further research is required to ascertain the structural folding of A-SAA when exerting its FPR2-mediated functions. Allosteric modulation of FPR by ligands other than A-SAA has been discussed in this review (*vide infra*).

### 5.2 A-SAA and A-SAA-derived peptides as FPR2-targeted therapeutic agents

Very interesting is the number of emerging reports that have delineated the role of A-SAA in orchestrating disease pathology through the KO of A-SAA in animal models of disease (6). For instance, Sano and colleagues described the role of SAA1/2 in regulating the T-helper 17 (Th17) cell-mediated immune response following colonization of mice with segmented filamentous bacteria (SFB). SAA1/2 was shown to not only upregulate the expression of IL-17A and IL-17F by Th17 cells but also to enhance the proliferation of IL-17-expressing RORγt^+^ Th17 cells (118). This is in agreement with the findings of Anthony et al. who demonstrated the role of the A-SAA-IL-17A axis in COPD *via* FPR2 (*vide supra*) (69). Moreover, the *in vivo* chemoattractant role of A-SAA was demonstrated within the context of apical periodontitis by Hirai et al. Indeed, following pulpal infection, SAA1/2 KO mice displayed a reduction in myeloid cell infiltration when compared to wild-type mice (119).

Although A-SAA is predominantly discussed in the literature as a pro-inflammatory mediator, a growing body of research suggests that a rather dual function is relayed by A-SAA during inflammation. For instance, Murdoch et al. showed that despite being essential for the maturation and recruitment of neutrophils in zebrafish, SAA also serves to confine neutrophil-mediated inflammation by reducing neutrophil bactericidal activity and the expression of inflammatory markers (120). Nonetheless, for the majority of these A-SAA-mediated roles, no receptor interaction has been assigned. In addition, as the biological characterization of A-SAA has been hampered by low-quality commercial A-SAA preparations (*vide supra*), the biological function of A-SAA is yet to be accurately elucidated. As such, further exploration of the A-SAA-FPR2 interaction is likely to yield insight into the role of this interaction in disease pathology. Indeed, due to its promiscuous cellular expression profile, FPR2 is linked to various pathophysiological processes mainly through its role in cellular chemotaxis. Moreover, its capacity to recognize structurally diverse ligands has implicated FPR2 in the pathology of diseases displaying variable underlying etiologies (121–123). Apart from its role in disease pathology, FPR2 is also described to instigate the resolution of disease (124). Hence, FPR2 might serve as a potential drug target. GPCRs constitute the largest class of druggable proteins within the human genome and remain a promising target for future drug development (125). Indeed, pharmaceuticals targeting GPCRs account for approximately 35% of FDA-approved drugs (126). Targeting chemoattractant GPCRs has been successful in some cases. For instance, Maraviroc, an allosteric modulator of the chemokine receptor CCR5, is currently marketed as an anti-HIV agent (105). Taken together, this leads us to the question whether A-SAA-derived peptides may serve as potential FPR2-targted therapeutic agents. Peptide drugs offer certain advantages. They display higher specificity and efficacy and lower immunogenicity and are hence safer with a rather tolerable side effect profile. Moreover, peptide drugs are cheaper to produce than, e.g. monoclonal antibodies. One major drawback observed with peptide drugs is the lack of *in vivo* stability conferred by secondary and tertiary structures leading to poor bioavailability. Nonetheless, extensive research continues to be carried out to produce peptide-based drugs with optimal pharmacokinetics (127).

**Figure 1:**
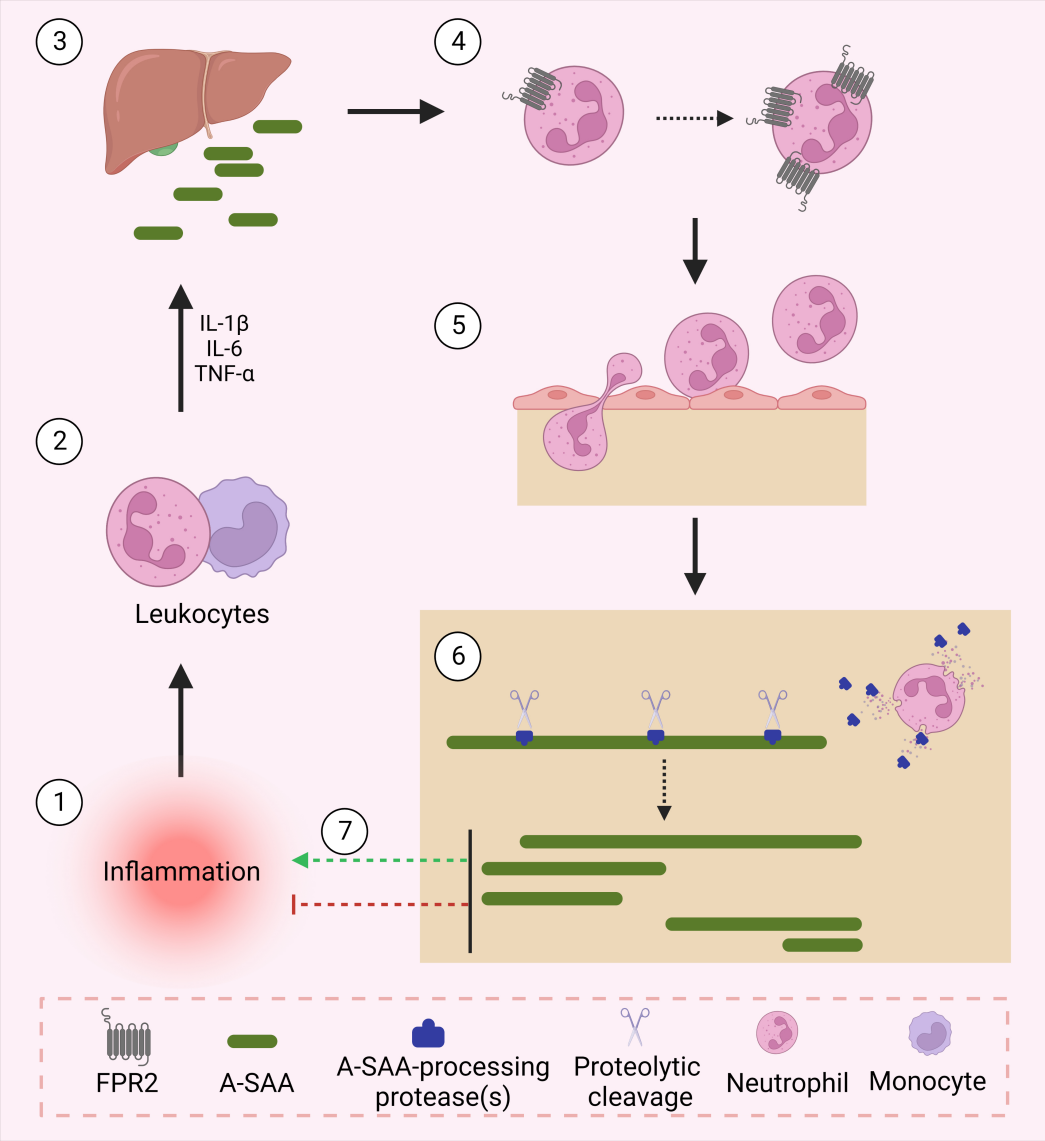
A-SAA and its derived peptides regulate inflammation. Following an inflammatory stimulus, leukocytes activate the acute phase response through their expression of cytokines namely, IL-1β, IL-6 and TNF-α. In turn, upregulated cytokines activate the liver to express acute phase proteins including A-SAA. A-SAA acts on neutrophils to upregulate their expression of the plasma membrane receptor FPR2. Through its activation of FPR2, A-SAA enhances the migration of neutrophils into the site of inflammation. At the site of injury, recruited neutrophils release proteases that cleave A-SAA at different sites thereby generating distinct A-SAA-derived peptides which either further enhance the inflammatory response or rather relay a pro-resolving function.

The identification and characterization of A-SAA-derived peptides is certainly not yet fully accomplished. We hypothesize that under inflammatory conditions, upregulated proteases process A-SAA, thereby generating a range of peptides, which display variable functions to modify the inflammatory reaction (**Figure 1**). As mentioned, SAA1.1-derived C-terminal peptides have been shown to magnify chemokine-induced chemotactic responses *via* their binding to FPR2 (*vide supra*). As such, further research is warranted to identify novel A-SAA-derived peptides, which could be further developed as drugs following their biological characterization. For instance, Jana et al. demonstrated the capacity of SAA1(1–5) to destabilize SAA fibrils, thereby placing the peptide as a promising lead drug for the treatment of amyloid A amyloidosis, a common secondary disease occurring within the context of uncontrolled inflammation (101).

Concerning FPR2, a growing body of reports have discussed its role as a potential therapeutic target using peptide-based drugs. The FPR2 ligand CGEN-855A, a 21 amino acid peptide, was demonstrated to provide an anti-inflammatory effect in a zymosan-induced air pouch model of inflammation and provided cardioprotection against ischemia reperfusion-induced injury *via* reduced PMN infiltration to the injured organ (128). CGEN-855A is reported to have reached preclinical development (124). Moreover, in a recent review by Ma et al., the orthosteric FPR2 agonistic hexapeptide, WKYMVm, has been discussed as a potential therapeutic to treat inflammation, cancer, angiogenesis, tissue repair, neurodegenerative diseases, vaccine development, insulin resistance and osteolytic diseases (83).

Despite the clinical success of orthosteric GPCR ligands, one major issue observed with such therapeutics remains to be the high degree of homology in the orthosteric binding sites of closely associated GPCRs thereby leading to off-target side effects. On this account, current research is directed towards the design and development of allosteric modulators of GPCRs (129). One assumed outcome of investigating A-SAA-derived peptides and their link to FPR2 activity is the identification of novel allosteric modulators of FPR2. The notion of allosteric modulation of FPR2 by peptides has been previously discussed in the literature. Zhang et al. demonstrated biased allosteric modulation of FPR2 by Ac_2-26_, an N-terminal peptide of AnxAI, wherein the AnxAI-derived peptide-induced FPR2 conformational changes that are thought to lower the binding and to inhibit the pro-inflammatory effect induced by WKYMVm. In sharp contrast, Aβ_42_, a peptide detected in the neuritic plaques in Alzheimer’s disease, brought about conformational changes that improved the binding affinity of WKYMVm hence exacerbating its pro-inflammatory function (130).

Pepducins represent a promising class of molecules that enable the study of GPCRs and have therapeutic potential. Pepducins are lipopeptides with an N-terminal lipid, typically, palmitate, linked to a short peptide wherein the peptide sequence is derived from the intracellular loop of the GPCR that it is presumably thought to target. The lipid moiety instigates the translocation of the pepducin across the plasma membrane where the pepducin functions to modulate receptor signaling (131). F2Pal_16_, derived from the third intracellular loop of FPR2, was demonstrated to activate neutrophils leading to enhanced inflammatory function (132). Nonetheless, the notion that pepducins will selectively target GPCRs from which they are derived does not always apply. In a report by Winther et al., an FPR1-derived pepducin, F1Pal_16_ demonstrated an inhibitory effect on FPR2-mediated neutrophil inflammation in response to a diverse set of ligands (MMK-1, WKYMVm, F2Pal_10_ and PSMα2) specific to the receptor suggesting that the pepducin displays a general inhibitory effect on FPR2. Whereas F1Pal_16_ was shown to compete with conventional FPR2 agonists, namely WKYMVm, for binding, it is currently unclear whether the inhibitory effect is mediated *via* competitive binding or rather through allosteric modulation inducing conformational changes that reduce the binding of the conventional agonist (133). Moreover, a protease-activated receptor (PAR) 4-derived pepducin, P4Pal_10_, was found to inhibit FPR2-mediated Gαi signaling in neutrophils following stimulation with WKYMVm. In addition, the pepducin did not compete with WKYMVm suggesting that its inhibitory effect is not relayed *via* direct interaction with the conventional agonist binding site (134). Finally, allosteric modulation of FPR2 on neutrophils using pepducins has also been discussed as a potential antibacterial strategy to solve the ongoing issue of antibiotic resistance (135).

## 6 Discussion

Although the SAA family has been identified approximately 5 decades ago, this APP remains highly enigmatic. While this is in part because the study of A-SAA has been greatly hindered by the use of contaminated recombinant variants, additional factors ascribed to the inherent structural nature of A-SAA have limited its investigation. Indeed, it is currently unclear how the intrinsically disordered nature of A-SAA and its propensity to form oligomers influences its capacity to activate FPR2, in turn regulating its functions in health and disease. Moreover, the investigation of A-SAA regarding its susceptibility to undergo PTM is currently limited. The effect of proteolytic processing on the biological activity of A-SAA has been scarcely studied and not any other PTM such as citrullination, nitration or glycosylation has been discovered. As such, further investigation is warranted to identify novel PTMs and their influence on the biological function of A-SAA. Not only will delineating the structure-activity relationship of A-SAA give insight into its biological function, but it will also serve as a framework to further understand the underlying mechanics of FPR2 activity presumably leading to novel therapeutic targets. Indeed, allosteric FPR2 agonists that can specifically shift the paradigm towards a more pro-resolving pathway represent a new therapeutic frontier.

## Author contributions

SAS wrote the initial draft. MG, SS, and JVD contributed to the manuscript conception and design, provided their comments on the manuscript and approved the final version of the manuscript.
